# QuickStats

**Published:** 2014-11-28

**Authors:** FROM THE NATIONAL CENTER FOR HEALTH STATISTICS

**Figure:**
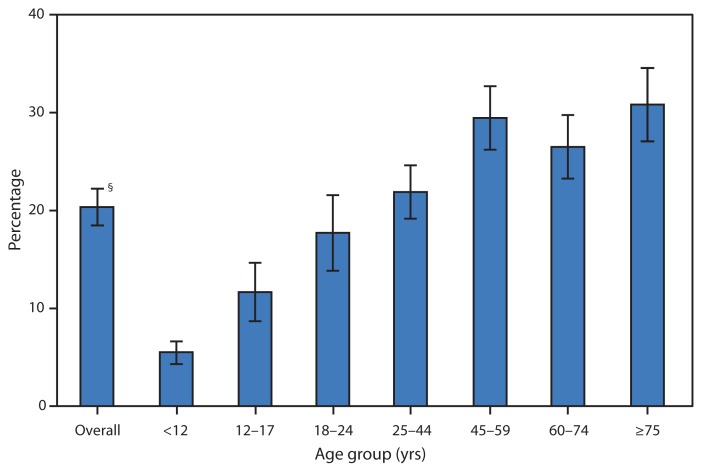
Percentage of Mental Health–Related^*^ Primary Care^†^ Office Visits, by Age Group — National Ambulatory Medical Care Survey, United States, 2010 ^*^ A mental health visit was defined by at least one of the following: ordering or provision of depression screening, psychotherapy, or other mental health counseling; a mental health diagnosis or reason for visit; or a psychotropic medication that was ordered, supplied, administered, or continued at the visit. Mental health diagnosis, reason for visit, and psychotropic medications were based on certain categories. Source: Olfson M, Kroenke K, Wang S, Blanco C. Trends in office-based mental health care provided by psychiatrists and primary care physicians. J Clin Psychiatry 2014;75:247–53. ^†^ Includes physicians in primary care specialties: general and family practice, internal medicine, pediatrics, and obstetrics/gynecology. ^§^ 95% confidence interval.

In 2010, 20% of all visits to primary care physicians included at least one of the following mental health indicators: depression screening, counseling, a mental health diagnosis or reason for visit, psychotherapy, or provision of a psychotropic drug. The percentage of mental health–related visits to primary care physicians increased with age through age 59 years and then stabilized. Approximately 6% of visits for children aged <12 years and approximately 31% of visits for adults aged ≥75 years were associated with mental health care.

**Source:** 2010 National Ambulatory Medical Care Survey. Available at http://www.cdc.gov/nchs/ahcd.htm.

**Reported by:** Donald K. Cherry, MS, dcherry@cdc.gov, 301-458-4762; Susan M. Schappert, MA.

